# Transfer of Immunity from Mother to Offspring Is Mediated via Egg-Yolk Protein Vitellogenin

**DOI:** 10.1371/journal.ppat.1005015

**Published:** 2015-07-31

**Authors:** Heli Salmela, Gro V. Amdam, Dalial Freitak

**Affiliations:** 1 Centre of Excellence in Biological Interactions, University of Helsinki, Helsinki, Finland & University of Jyväskylä, Jyväskylä, Finland; 2 School of Life Sciences, Arizona State University, Tempe, Arizona, United States of America; 3 Department of Chemistry, Biotechnology, and Food Science, Norwegian University of Life Sciences, Aas, Norway; Stanford University, UNITED STATES

## Abstract

Insect immune systems can recognize specific pathogens and prime offspring immunity. High specificity of immune priming can be achieved when insect females transfer immune elicitors into developing oocytes. The molecular mechanism behind this transfer has been a mystery. Here, we establish that the egg-yolk protein vitellogenin is the carrier of immune elicitors. Using the honey bee, *Apis mellifera*, model system, we demonstrate with microscopy and western blotting that vitellogenin binds to bacteria, both *Paenibacillus larvae* – the gram-positive bacterium causing American foulbrood disease – and to *Escherichia coli* that represents gram-negative bacteria. Next, we verify that vitellogenin binds to pathogen-associated molecular patterns; lipopolysaccharide, peptidoglycan and zymosan, using surface plasmon resonance. We document that vitellogenin is required for transport of cell-wall pieces of *E*. *coli* into eggs by imaging tissue sections. These experiments identify vitellogenin, which is distributed widely in oviparous species, as the carrier of immune-priming signals. This work reveals a molecular explanation for trans-generational immunity in insects and a previously undescribed role for vitellogenin.

## Author Summary

Insects lack antibodies, the carriers of immunological memory that vertebrate mothers can transfer to their offspring. Yet, it has been shown that an insect mother facing pathogens can prime her offspring’s immune system. To date, it has remained enigmatic how insects achieve specific trans-generational immune priming despite the absence of antibody-based immunity. Here, we show this is made possible via an egg-yolk protein binding to immune elicitors that are then carried to eggs. This yolk protein, called vitellogenin, is able to bind to different bacteria and pathogenic pattern molecules. We use *E*. *coli* fragments as a bait to show how vitellogenin is necessary for the carrying of immune elicitors to eggs. These findings help to understand how insects fight pathogens and can be useful for protection of ecologically and economically important insects, such as the honey bee, that we used as a model species.

## Introduction

Insects lack antibodies, the carriers of immunological memory in vertebrates. Therefore, it has been thought that insects are deprived of acquired immunity and only have innate defense mechanisms against pathogens. Recent research, however, has shown that insects are capable of high specificity in their defense reactions; indeed, insect immune defenses can recognize specific pathogens [1] and prime offspring against them [2,3].

Immunity is a major mechanism of survival that carries significant physiological and energetic costs, thus, immune responses must be regulated to maximise fitness [4,5]. Immunocompetence is traded-off against other life-history traits, such as growth and development, when the risk of infection is low. In order to maximize the fitness of their offspring in terms of immunity, growth rate and reproductive potential, selection should favour passing on a plastic signal (i.e. presence or absence of pathogens) about the pathogenicity of the environment. It has been observed that many organisms can transfer highly specific immune protection to the next generation [6].

Trans-generational immune priming (TGIP) was initially attributed to animals with antibody-based adaptive immune systems [6]. The discovery that invertebrates, equipped only with innate immune responses, are also able to prime their offspring against infections has changed the understanding of innate immunity. Interestingly, even nonpathogenic bacteria in diet can trigger systemic immune responses in both the same generation and in the next [7,8]. Cumulative evidence shows how maternal exposure to immune elicitors, and dead or living bacterial cells, leads to higher immunocompetence in the offspring [8–12]. For example, Moret et al. (2006) found increased immunity in the next generation after injecting adult mealworm (*Tenebrio molitor*) females with bacterial lipopolysaccharides (LPS) [9]. Also, in the red flour beetle (*Tribolium castaneum*), Roth et al. (2009) showed that parental exposure to the Gram-positive soil-dwelling bacterium, *Bacillus thurngiensis*, could elicit strain-specific TGIP [10], while Freitak et al. (2009) found that feeding non-pathogenic bacteria to female cabbage loopers (*Trichoplusia ni*) during larval stage resulted in higher steady state immunity in the next generation [8]. In the tobacco hornworm (*Manduca sexta*), Adbel-latief & Hilker (2008) demonstrated that the embryonic immune system is up-regulated after injection of immune elicitors into eggs [13]. Finally, Hernandez-Lopez et al. (2014) showed that injecting honey bee (*Apis mellifera*) queens with dead *Paenibacillus larvae* (bacterium responsible for the American foulbrood disease) leads to higher resistance against this pathogen in the offspring [14]. These findings have created a central dilemma in immunological physiology regarding how immune priming can be mediated by mechanisms other than antibodies.

Innate and adaptive immune responses are triggered by pathogen-associated molecular patterns, or immune elicitors. Immune elicitors are present on the cell walls of bacteria and fungi [1]. TGIP appears to be mediated by fragments of such pathogenic microorganisms, which can be transferred from insect midgut lumen to the hemocoel [2]. In the hemocoel, fragments are transferred and incorporated into fat body, a tissue that is functionally homologous to liver and white adipose tissue in vertebrates. Eventually, fragments are detected in developing eggs [2]. These findings suggest that microbial fragments are transferred from mother to offspring, carrying specific immune elicitors to mediate appropriate immune responses. However, it has remained unknown exactly how the immune elicitors can enter insect eggs.

The ability to utilize TGIP mechanisms can be of considerable economic importance. Industries that rely on beneficial invertebrates can develop methods of prevention against contagious diseases, whereas industries dealing with pest control can instead induce reduced TGIP. One invertebrate that can benefit from a commercial utilization of TGIP is the honey bee, *Apis mellifera*. The honey bee is an ecologically and economically important pollinator of many wild plants as well as cash crops. At the same time, it is susceptible to many diseases, and thus like many other important pollinators, is in global population decline. Since TGIP was recently confirmed in the honey bee system, i.e. in response to the pathogen responsible for American foulbrood disease, we here combined biochemistry and histology to trace the fate of the immune elicitors during insect egg development under threat of pathogens.

We hypothesized that TGIP is mediated by a protein that plays roles both in egg-yolk formation and immunity–vitellogenin (Vg). Vg is a yolk precursor as well as a pathogen pattern recognition receptor [15]. It is a nutritious lipoprotein synthesized by the fat body or vertebrate liver, secreted to the hemolymph/blood and taken up by nurse cells and eggs by receptor-mediated endocytosis [7]. Vg concentration varies between the members of the honey bee colony, from almost undetectable to 40% of the total hemolymph protein fraction in the functionally sterile helper females, called workers—and it constitutes up to 70% of the hemolymph protein fraction in the egg-laying queens [16]. In fish, Vg binds to LPS of Gram-negative bacteria, to peptidoglycan (PG, a major constituent of the cell-wall of Gram-positive bacteria), and to surface glucan of fungi [17]. These immunological properties of Vg are little explored in species other than fish. Here, we reveal how honey bee Vg has similar immunological binding properties, and, for the first time, demonstrate how the bound immune elicitors can enter eggs via Vg uptake to the ovary. These results suggest a central role for Vg in TGIP.

## Results

### 1. Vg binds to bacteria and pathogen patterns

**Fig 1:**
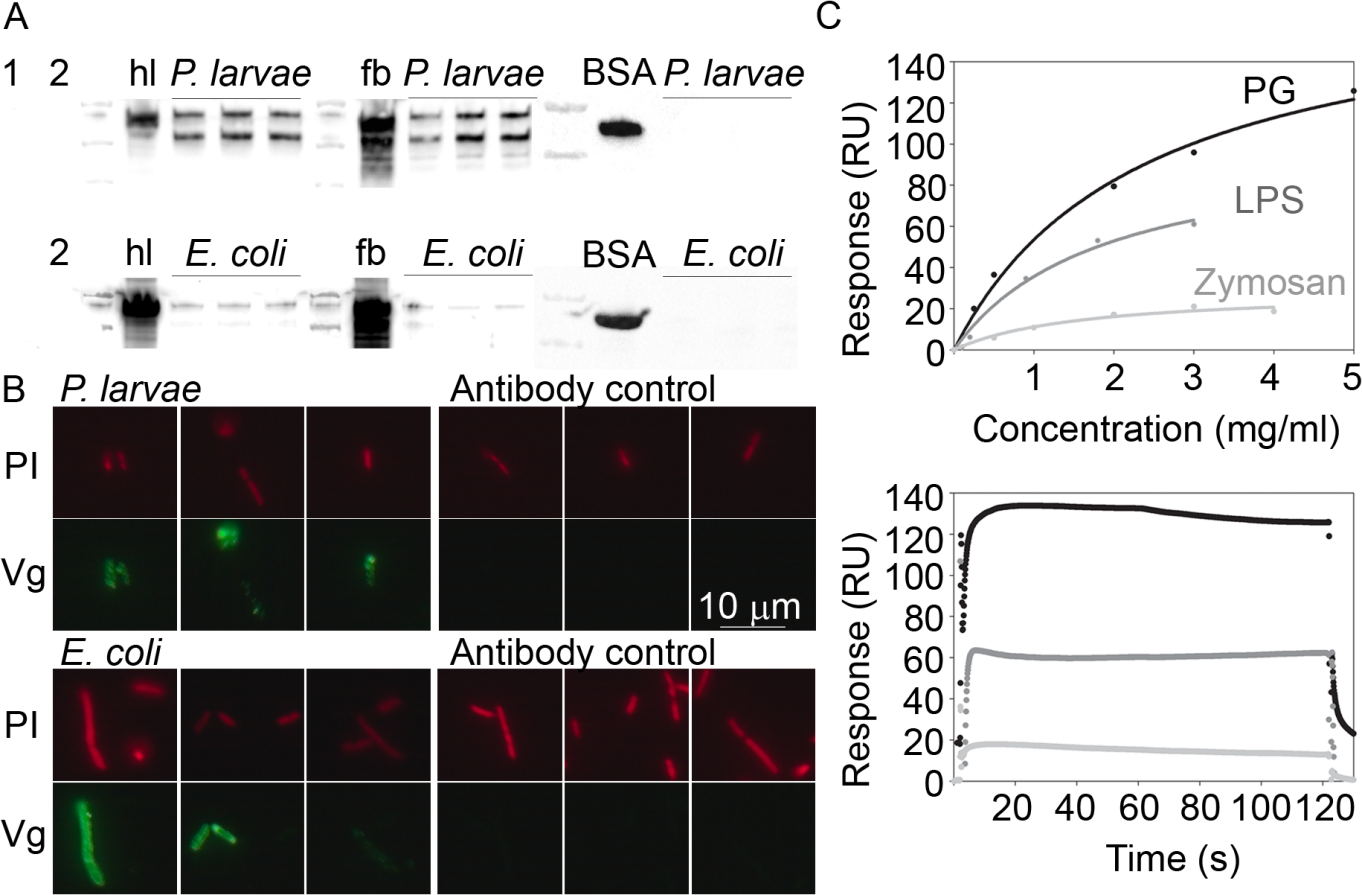
Honey bee Vg binding to bacteria was tested by western blotting (A) and microscopy (B). Binding to candidate pathogenic molecules was further tested by surface plasmon resonance technique (C). (A) Vg-rich honey bee hemolymph (hl), Vg-rich fat body protein extract (fb), or bovine serum albumin control (BSA) were incubated with *P*. *larvae* or *E*. *coli*, after which the bacteria were washed and blotted using an antibody that detects Vg or BSA. Untreated control samples are indicated (hl, fb and BSA), and next to them are located the bacteria-incubated test samples (N = 3) marked with an overhead line. Two negative controls are numbered: 1 = Control for Vg aggregation (fb without bacteria), and 2 = control for unspecific antibody binding to *P*.*larvae* (above) or *E*.*coli* (below). The image exposure time for the *P*. *larvae* blot was 1 s, and 5 s for the *E*. *coli* blot to better reveal its weaker bands. Vg appears as a double band of 180 and 150 kDa. (B) Representative images of *P*. *larvae* and *E*. *coli* that were incubated with hemolymph, and carefully washed and fixed (N = 3). The bacteria were visualized using propidium iodide (PI; red). Vg was detected using an Alexa fluor 488 nm conjugated secondary antibody (green). The primary antibody was omitted in the secondary antibody control. (C) PG, LPS and zymosan binding to Vg immobilized on a surface plasmon resonance chip. The data are blank subtracted. Above: The X-axis shows the analyte concentration, and the Y-axis shows the binding response. The curves were fitted based on five measurements at different analyte concentrations. The dots mark the binding response at each concentration measurement point. Below: The sensogram data of the maximal concentration for each analyte.

We first verified that honey bee Vg can bind to *P*. *larvae*–the Gram-positive bacterium that causes American foulbrood disease–and to Gram-negative *E*. *coli* by using western blotting and microscopy with live bacteria and an antibody that recognizes Vg (Fig 1A and 1B). In the western blot, Vg signal was found in both *P*. *larvae* and *E*. *coli* samples that had been incubated with Vg-rich honey bee hemolymph or fat body homogenate and then thoroughly washed (Fig 1A). The Vg signal appears to be stronger in the *P*. *larvae* samples than in the case of the *E*. *coli* samples. Negative controls were used to verify that the Vg signal was not due to Vg aggregation (a sample of fat body homogenate without any bacteria; lane 1, Fig 1A) or due to unspecific antibody binding to bacteria (samples of bacteria only; lanes numbered 2, Fig 1A). Also, bovine serum albumin (BSA) was used as a negative control, and this protein showed no binding to either bacterial species (Fig 1A; BSA). We did fluorescence microscopy of *P*. *larvae* and *E*. *coli* incubated with honey bee hemolymph to verify the western blot result, and Vg signal was observed covering the bacteria (Fig 1B). The antibody controls for unspecific binding showed no signal.

We then verified honey bee Vg binding to the pathogen patterns PG (predominantly a Gram-positive bacteria signature molecule), LPS (Gram-negative signature) and zymosan (yeast) using a surface plasmon resonance technique (Fig 1C). We detected the highest binding response for PG followed by LPS, whereas the binding response to zymosan was modest.

### 2. Vg is required for the transport of bacteria-derived molecules into eggs

**Fig 2:**
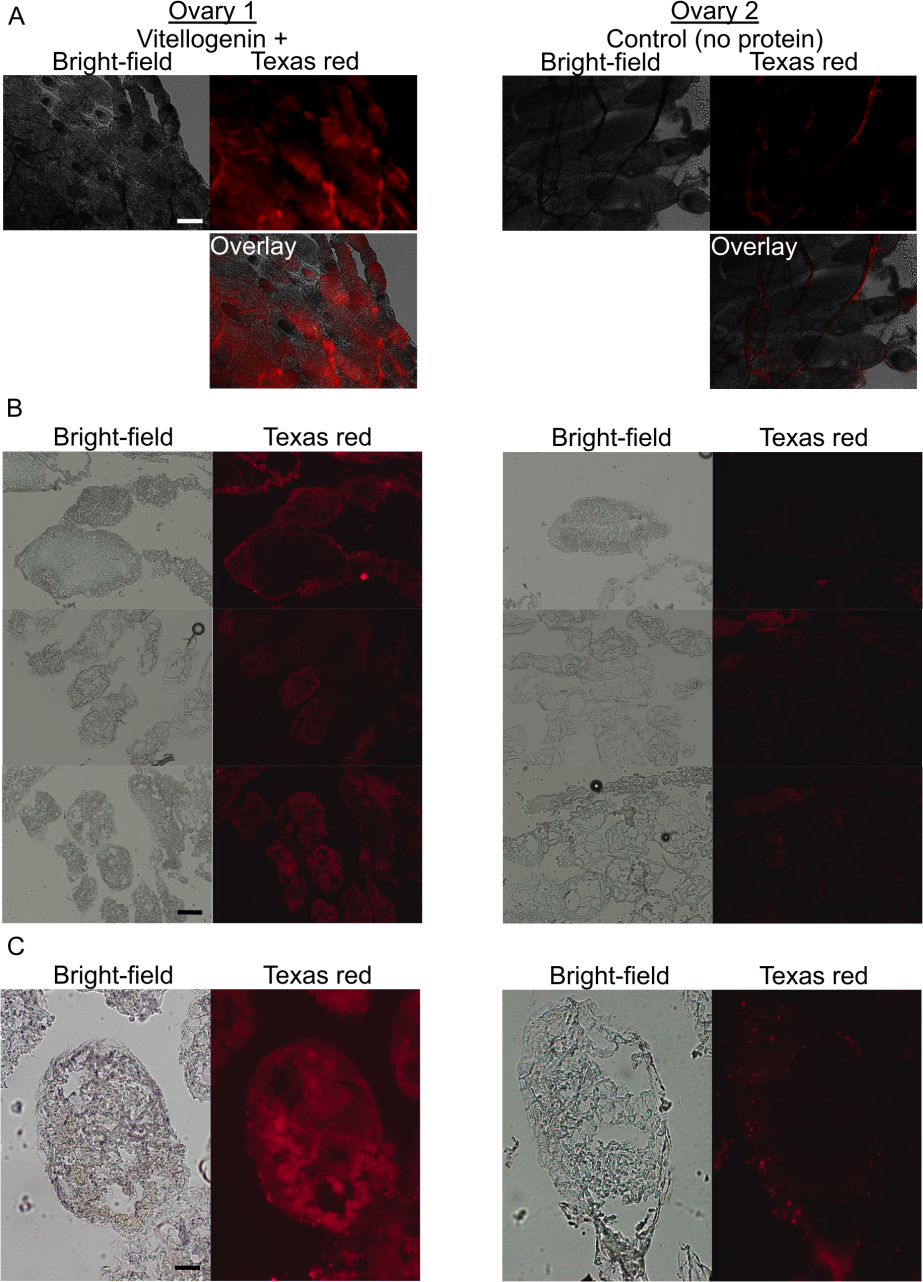
The localization of bacterial fragments in honey bee queen ovaries in the presence (left) and absence (right) of pure Vg. Freshly detached ovaries were incubated in buffer containing fluorescent (Texas red) *E*. *coli* fragments, and imaged right after (A) or embedded for cryo-sectioning and imaging later (B-C). (A) Whole ovaries mounted and imaged immediately after incubation and washing steps. 5 x magnification, the scale is 200 μm. (B) Eggs in cryo-sectioned ovaries. 10 x magnification, the scale is 200 μm. (C) A single egg in a cryo-sectioned ovary; 20 x magnification, the scale is 50 μm. In the Vg-incubated ovaries, eggs with internalized fluorescent material were observed. In the control (right), the bacterial fragments were, typically, found as bright aggregates on the membranes surrounding the eggs. The images represent N = 6 queens.

Next, we verified that Vg can carry pathogen-derived molecules into eggs. This was tested by incubating dissected honey bee queen ovaries with the commercially available fluorescently labeled *E*. *coli* fragments, followed by imaging the fluorescent material taken up by the ovarioles (ovarian filaments) in the absence and presence of purified Vg (Fig 2). The uptake of bacterial material was found only in the eggs that were provided with Vg. This result is consistent with our proposition that Vg is a carrier of TGIP messages.

### 3. Vg is sufficient and necessary for TGIP

**Fig 3:**
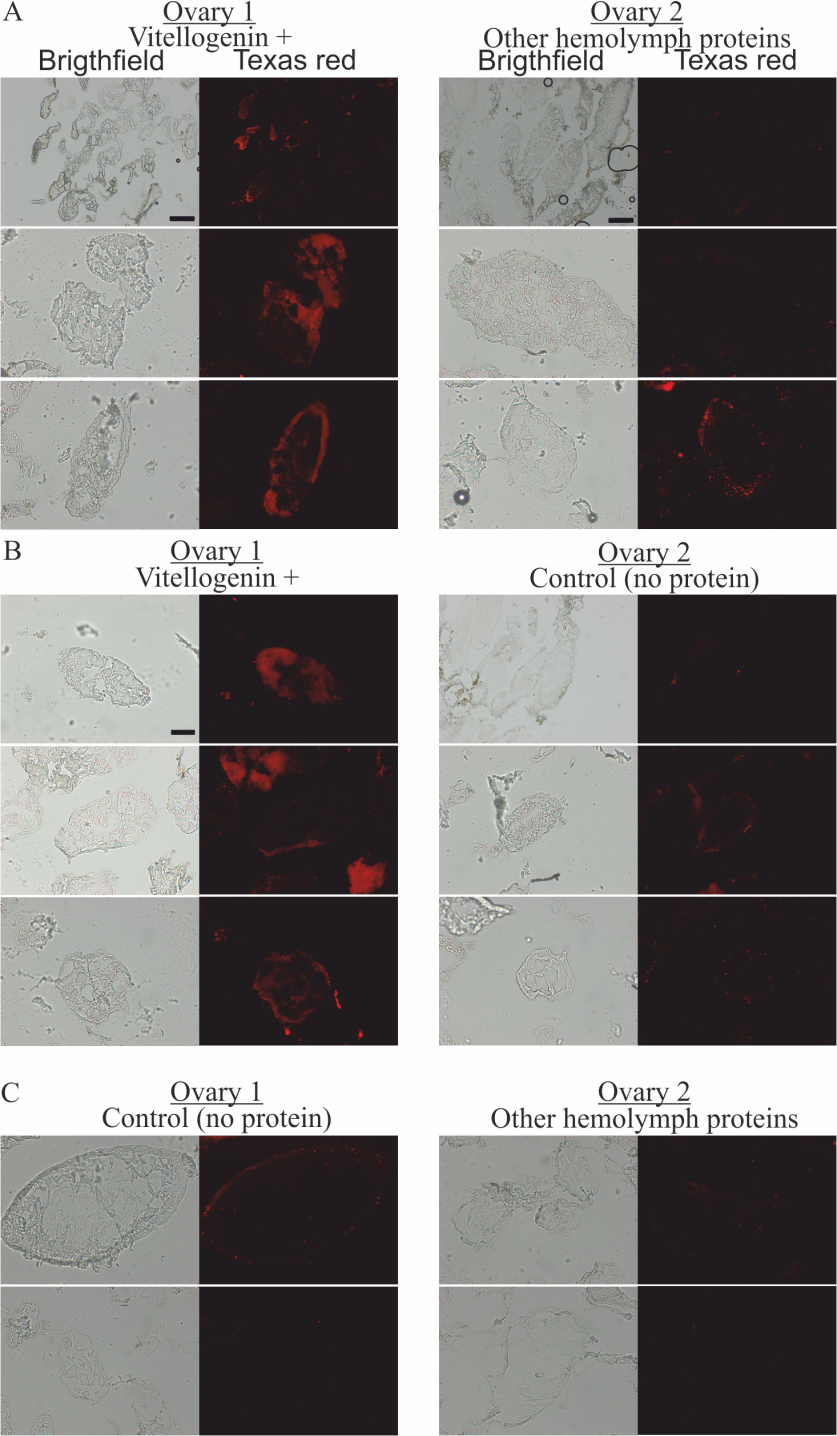
The localization of fluorescently-labeled bacterial fragments in cryo-sectioned honey bee queen ovaries incubated in the presence of pure Vg, in the presence of hemolymph proteins other than Vg, and in the absence of any externally provided protein. (A) One ovary was incubated with Vg (left) and the other with other hemolymph proteins (right), N = 3. (B) One ovary was incubated with Vg (left) and the other without any protein (right), N = 3. (C) One ovary was incubated without any protein (left) and the other with other hemolymph proteins but Vg (right), N = 2. The scale is 50 μm, or 200 μm (the latter is indicated with scale bars).

Finally, Vg was found to be a sufficient and necessary hemolymph protein for the transfer of immune elicitors to occur. To show this, we tested if the presence of other, non-Vg honey bee hemolymph proteins produced by ion-exchange fractioning of honey bee hemolymph can trigger the transfer of immune elicitors to the developing eggs. The major protein fractions in the samples of other proteins are apolipophorin and hexamerins that are involved in transport and storage functions (see S1 Fig for an SDS-PAGE gel of hemolymph, pure Vg and the other non-Vg proteins, and a hemolymph fractioning chromatogram). In the case of the non-Vg hemolymph proteins, the result was negative (Fig 3).

## Discussion

We establish here a previously undescribed role for the major egg-yolk precursor protein Vg as the carrier of immune elicitors from mother to eggs in insects. Using the honey bee as a model, we first confirm that Vg binds to different types of bacteria, both *P*. *larvae*, a Gram-positive pathogen that infects and kills honey bee larvae, and to *E*. *coli* that represents Gram-negative bacteria. This binding could not be mimicked by BSA. Next, we verify that Vg binds to pathogen-associated molecular patterns. Finally, we document that Vg is required for the transport of fluorescently labeled cell wall pieces of *E*. *coli* into developing eggs in ovaries. These experiments show for the first time that Vg serves as a carrier of immune-priming signals. This finding provides a new molecular mechanism behind trans-generational immunity in oviparous species.

Although not conclusive, our bacteria western blot with stronger Vg binding to *P*. *larvae* than *E*. *coli* and surface plasmon resonance data with stronger PG binding response compared to LPS hint that Vg might have a binding preference to Gram-positive bacteria. This could be an adaptation to the major bacterial threats of the honey bee larvae: *P*. *larvae* **,** as well as *Melissococcus plutonius* which causes European foulbrood disease. These pathogens are both Gram-positive bacteria.

Several human lipoproteins bind to a broad range of hydrophobic inflammatory molecules including bacterial surface structures and remnants of necrotic cells in an anti-inflammatory manner [18,19]. Based on our current and previous data, we propose that the insect lipoprotein Vg shares a similarly broad binding range. We previously found that honey bee Vg binds strongly to phosphatidylserine containing liposomes, to blebs of apoptotic insect cells and to necrotic cells packed with phosphatidylserine, while having modest binding capability to healthy insect cell membrane or liposomes with neutral phosphatidylcholine [20]. The negative charge of phosphatidylserine may explain the selectivity of Vg binding between lipids, as Vg α-helical part seems to have higher affinity towards negatively charged damaged cell membranes [20]. We speculate that the combination of negative charge and hydrophobicity can provoke Vg binding to the bacterial PG and LPS signature molecules as well.

Vg participation in TGIP can represent a co-option of the protein’s dual role in fecundity and immunity [21]. The gene (*vitellogenin*) experiences rapid evolution in the honey bee [22], it is present in different copy numbers in different insect species [23], and has several homologous genes in some insects [24]. Mutation hotspots are found within the honey bee *vitellogenin* sequence, and the multiple alleles are under ongoing positive selection in Africa, East- and West-Europe. By analogy to vertebrate adaptive immunity [15,25,26], certain Vg variants could be more sensitive to specific pathogen recognition. *Vitellogenin* alleles in at least some insects may thus evolve under local pathogen pressure. We speculate that changes in pathogen pressure over time and in different environments are reflected in these interesting patterns of *vitellogenin* evolution.

Examining the roles of Vg in invertebrate TGIP can open up entirely new areas in immunology. Immune responses can be very specific and induced by pathogen associated molecular markers present on the cell walls of microorganisms (PG, LPS, surface glucans). TGIP can occur and disappear very rapidly, is often maternally transmitted and shows pathogen specificity. The new discovery of a Vg-mediated transfer-mechanism, as described here, would be consistent with all these observations.

For Vg-mediated TGIP to occur, the mother must be exposed to a certain amount of pathogenic cell wall fragments during or immediately prior to reproduction. Bacteria are actively lysed in the gut lumen by the digestive system, as well as in the hemolymph by the immune system. Once in the hemolymph, the immune elicitors are available for binding to Vg and for transfer to the developing eggs in the ovary. This route would allow a mother to prime her offspring against the specific infections present in her current environment. When the environment becomes pathogen-free and infection has cleared from the adult female, no transfer to her eggs would take place. In this manner, the cost of resistance to infections in offspring would be avoided.

We propose that Vg-mediated TGIP can allow for efficient, specific and environmental-dependent immune priming in insects. However, this mechanism does not rule out that other mechanisms also participate in TGIP. These can include paternal TGIP, other molecules transported by mothers or epigenetic modifications [27,28]. In this context, it is interesting to note that male insects also produce Vg, and that Vg can be found in their seminal fluids [29].

Vg-mediated transfer of pathogenically inactive bacterial fragments could provide a platform for the development of vaccines for beneficial insects. For example, pollinator-oriented medical genetics could aim to identify the most TGIP efficient *vitellogenin* alleles to improve honey bee colony survival. The reproductive female, the queen honey bee, is typically shielded from harsh environmental conditions and infection. However, her environment is never sterile. Exposure can occur by direct contact or by contaminated food, and honey bee queens are likely to experience some levels of pathogen load [30–32]. Conversely, knowledge about Vg-mediated TGIP can also open the door for modifying or hijacking TGIP in pest insect species. For example, TGIP may be impaired by chemically modifying the binding properties of Vg. Alternatively, TGIP could be exploited to trigger a reaction against the pathogenic insect’s symbionts, or to put the immune system in overdrive—increasing the cost of immunity and reducing investment in reproduction. In sum, such applications could be highly beneficial in agriculture.

It remains to be tested whether Vg-mediated transfer of immune elicitors occurs in egg-laying vertebrates. If yes, then the vertebrate lineage would have retained an ancient TGIP mechanism in addition to their evolutionary innovation of transfer of antibodies.

## Materials and Methods

### 1. Western blot with live *P*. *larvae* and *E*. *coli*

Wintertime worker honey bee hemolymph (hl) and fat body protein extract (fb) are rich in Vg, and were used for testing Vg-binding to bacteria, adapted from the fish Vg experiment by Tong *et al*. [17] using an antibody that detects honey bee Vg. For cell-free hl and fb sampling, see Havukainen *et al*. [33]. The experiment was performed at room temperature, centrifugation steps were 3,000 g for 5 min, and wash volume was 0.5 ml of PBS, if not mentioned otherwise. *P*. *larvae* (strain 9820 purchased from Belgian Co-ordinated Collections of Micro-organisms, Gent, Belgium) grown on MYPGP agar plates for 7 days and Epicurian Gold *E*. *coli* grown in LB medium liquid culture overnight were washed and suspended in 100 μl PBS per sample. The bacteria suspensions (~1.3 x 10^8^ cells/ml) were mixed with either an equal volume of hemolymph diluted 1/10 in PBS with a protease inhibitor cocktail (Roche, Penzberg, Germany) or with fat body protein extract (5.7 mg/ml total protein in PBS with the protease inhibitors). The following negative controls were used: 1) 100 μl *P*.*larvae* and *E*. *coli* with an equal volume of PBS but no hl/fb, to detect possible unspecific antibody binding to the bacteria, 2) 100 μl fb with an equal volume of PBS, but no bacteria, to detect possible Vg aggregation, and 3) 100 μl *P*.*larvae* and *E*. *coli* treated with 100 μl 5 mg/ml bovine serum albumin (BSA; control protein). As untreated controls, we kept on ice 0.1 μl of hl, 0.5 μl of fb extract, and 1 μl of BSA. The samples were incubated at 26°C for 50 min under agitation for Vg-bacteria binding to occur. The bacteria were washed six times. The final pellet was resuspended in 10 μl of 4 M urea in PBS, agitated for 15 min and centrifuged. The samples were blotted on a nitrocellulose membrane according to a standard horse-radish peroxidase conjugate protocol with the Vg antibody tested before [33,34] (dilution 1:25,000; Pacific Immunology, Ramona, CA, USA), or a commercial BSA antibody (1:2000; Life Technologies, Carlsbad, CA, USA). The bands were visualized using Immune-Star kit and ChemiDoc XRS+ imager. All blotting reagents were purchased from Bio-Rad (Hercules, CA, USA).

### 2. Microscopy of *P*. *larvae* and *E*. *coli*

Vg-binding to bacteria was further tested by fluorescence microscopy. The incubation with hl was as above, except hl and bacteria volumes were both 20 μl and the number of bacterial cells was ~3 x 10^6^. All centrifugation steps were 10,000 g, +4°C, 5 min and PBS-T wash volumes were 1 ml. After hl incubation with the bacteria, the bacteria were washed and fixed with 4% paraformaldehyde for 10 min in room temperature. The cells were washed twice and blocked with 5% milk in PBS-T for 30 min in room temperature and washed again. Vg primary antibody (same as above) was used 1:50 in PBS-T and 1% milk for overnight incubation at +4°C. The samples were washed twice and incubated with Alexa fluor 488 nm anti-rabbit antibody, 1:50, for 1 h in room temperature in dark and washed three times. DNA was stained with standard propidium iodide (PI) protocol (Invitrogen). The bacteria were mounted with glycerol and imaged with Zeiss Axio Imager M2, excitations 499 nm and 536 nm, and emissions 519 nm and 617 nm. The primary antibody was omitted in the treatment of the secondary antibody control samples.

### 3. Surface plasmon resonance with LPS, PG and zymosan

Vg was purified from honey bee hemolymph with ion-exchange chromatography as described before [20,34]. Biacore T200 instrument (GE Healthcare, Waukesha, USA) and buffers from the manufacturer were used. The analytes were bought from Sigma Aldrich: PG from *S*. *aureus* #77140, LPS from *E*. *coli* #L2630 and zymosan from *S*. *cerevisiae* #Z4250. 30 μl/ml Vg in 10 mM acetate buffer pH 4.5 was immobilized on a CM5 chip—primed and conditioned according to the manufacturer’s instructions—until the response reached 5150 RU. The chip was blocked using ethanolamine. The analytes were suspended in the running buffer (0.1 M HEPES, 1.5 M NaCl and 0.5% v/v surfactant P20) and heated at 90°C for 30 min with repeated vigorous vortexing, followed by spinning in a table centrifuge for 20 min. Zymosan was heated for an additional 30 min at 95°C before centrifugation. PG and zymosan form a fine suspension in water solutions, and they formed a pellet during the centrifugation; their concentrations are given here as the weight added to the volume. The analytes were run with 120 s contact time and 600 s dissociation time with a 30 μl/min flow rate at 25°C. The analytes flowing in a separate channel on a naked chip was used as a blank, whose value was subtracted from the sample. After optimizing the binding-range, the following concentrations were measured. PG: 0, 0.25, 0.5, 2, 3, 5 mg/ml; LPS: 0, 0.1, 0.2, 0.9, 1.8, 3 mg/ml, and zymosan: 0, 0.5, 1, 2, 3, 4 mg/ml. PG and LPS binding did not reach binding saturation, yet, we did not exceed 5 mg/ml or 3 mg/ml concentration, respectively, to avoid analyte aggregation (see the manufacturer’s information and references therein for work concentrations).

### 4. Microscopy of queen ovaries

Six one year old *A*. *mellifera ligustica* queens were anesthetized on ice. Their ovaries were dissected and washed in ice cold PBS. One of the paired ovaries per queen was then placed in control solution (50 μl PBS containing 2 mg/ml Texas Red labeled *E*. *coli* Bioparticles; Life Technologies, Carlsbad, CA, USA) and the other ovary was placed in the same solution that contained, in addition, 0.5 mg/ml Vg purified from honey bee hemolymph [20,33]. The ovaries were incubated at 28ºC for 2 h under agitation. Next, the ovaries were washed twice in 1 ml ice cold PBS for 5 min under agitation. Samples of two queens were directly mounted using Fluoromount (Sigma) and observed by bright field and fluorescence (excitation 595 nm, emission 615 nm) microscopy (Axio Imager M2, Carl Zeiss AG, Oberkochen, Germany). One additional untreated control queen was imaged for detection of the autofluorescent pedical area of the ovary. The remaining four queens were embedded in Tissue-Tek (Sakura Finetek, Torrance, CA, USA) and stored in -80ºC. These ovaries were cut in 17 mm sections at -20ºC, and imaged immediately after mounting. The microscopy settings were kept constant during imaging.

To test whether hemolymph proteins could trigger the uptake of immune elicitors even in the absence of Vg, we modified the experimental setup to include hemolymph proteins other than Vg, the majority of which are apolipophorin and hexamerins, both known to bind to immune elicitors [35]. The other hemolymph proteins were obtained by running ion-exchange chromatography on honey bee hemolymph and dividing the collected hemolymph fractions into Vg and non-Vg proteins (S1 Fig) [20,33]. Remaining small molecular weight hemolymph molecules, such as possible peptides and hormones, were removed during protein concentration using centrifugal filters with 50 kDa cutoff with both Vg and non-Vg fractions (Millipore, Billerica, MA, USA). Fractions containing both Vg and other hemolymph proteins were discarded. The Vg and the non-Vg proteins had a final concentration of 0.5 mg/ml in the experiment. The queens were as above. The setup was as follows (all incubations contained the *E*. *coli* Bioparticles 1.5 mg/ml): one ovary was incubated with Vg and the other ovary with control solution (see above) (N = 3); one with Vg and the other with non-Vg hemolymph proteins (N = 3), and one ovary with non-Vg hemolymph proteins and the other with control solution (N = 2). The cryo-section imaging was done as above.

## Supporting Information

S1 Fig Chromatographic fractioning of honey bee hemolymph to Vg and other proteins. S = size standard. (A) An SDS-PAGE gel with a honey bee hemolymph sample used for protein fractioning. The major proteins are (in size order) apolipophorin, vitellogenin and hexamerins. (B) Pure vitellogenin and other hemolymph proteins produced by ion-exchange chromatography. The faint ~150 and ~40 kDa bands in the pure vitellogenin fraction are the previously mass-spectrometrically verified vitellogenin fragmentation products [33]. (C) Hemolymph fractioning chromatogram. The X-axis shows the time with 0.5 ml/min flow rate, and the Y-axis shows the percentage of 0.45 M NaCl phosphate buffer. The fraction collected as pure Vg is highlighted grey. The other protein fraction collected is indicated below the X-axis.
(JPG)
