# Supplementary figures and images for: Transfer of Immunity from Mother to Offspring Is Mediated via Egg-Yolk Protein Vitellogenin
Source: PLoS Pathog. 2015 Jul 31;11(7):e1005015. doi: 10.1371/journal.ppat.1005015 (PMC4521805; doi:10.1371/journal.ppat.1005015)

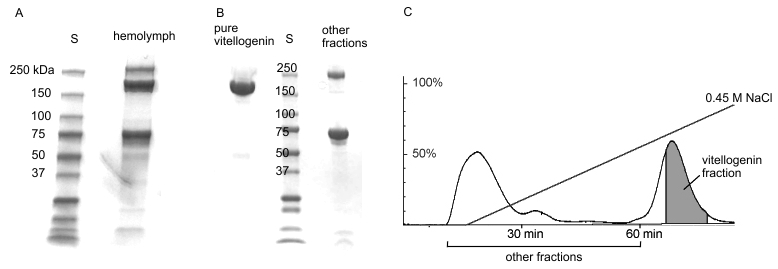

Supplement: S1 Fig — S = size standard. (A) An SDS-PAGE gel with a honey bee hemolymph sample used for protein fractioning. The major proteins are (in size order) apolipophorin, vitellogenin and hexamerins. (B) Pure vitellogenin and other hemolymph proteins produced by ion-exchange chromatography. The faint ~150 and ~40 kDa bands in the pure vitellogenin fraction are the previously mass-spectrometrically verified vitellogenin fragmentation products [33]. (C) Hemolymph fractioning chromatogram. The X-axis shows the time with 0.5 ml/min flow rate, and the Y-axis shows the percentage of 0.45 M NaCl phosphate buffer. The fraction collected as pure Vg is highlighted grey. The other protein fraction collected is indicated below the X-axis. (JPG) [file ppat.1005015.s001.jpg]
